# Support From Dementia Patients and Caregiver Burden: Exploring Variation by Caregiver Physical Health

**DOI:** 10.1093/geroni/igaf122.2532

**Published:** 2025-12-31

**Authors:** Xinyue Yang, Meng Huo, Kyungmin Kim, Dahua Wang

**Affiliations:** Beijing Normal University, Beijing, Beijing, China (People’s Republic); University of California, Davis, Davis, California, United States; Seoul National University, Seoul, Republic of Korea; Beijing Normal University, Beijing, Beijing, China (People’s Republic)

## Abstract

A small but growing body of work suggests that caregivers still receive support from people living with Alzheimer’s disease and related dementias (PLWD). Yet, we know little about what characteristics of the support received from PLWD benefit caregivers and whether the benefits apply to all caregivers. The current study extended prior research by examining (a) how the frequency and quality of the support that caregivers received from PLWD were associated with caregiver burden and (b) whether these associations varied by caregivers’ own physical health. We analyzed data from 72 spousal caregivers of older adults with mild-to-moderate Alzheimer’s disease. Caregivers reported their own and partners’ demographic characteristics, the frequency and quality of support they received from PLWD, caregiver burden, and their physical health. Multiple regressions showed that receiving emotional support more often and receiving better-quality support were both associated with lower caregiver burden. These associations, however, were stronger among caregivers who self-rated better physical health. This study showcases the benefits of receiving support from PLWD and emphasizes the importance of facilitating mutually beneficial relationships in dementia caregiving dyads. Further, our findings can inform tailored interventions targeting caregivers with different physical health conditions.

